# Nucleotide variation in Sabin type 3 poliovirus from an Albanian infant with agammaglobulinemia and vaccine associated poliomyelitis

**DOI:** 10.1186/s12879-016-1587-y

**Published:** 2016-06-10

**Authors:** Thomas Foiadelli, Salvatore Savasta, Andrea Battistone, Majlinda Kota, Carolina Passera, Stefano Fiore, Silvia Bino, Concetta Amato, Alessandro Lozza, Gian Luigi Marseglia, Lucia Fiore

**Affiliations:** Department of Pediatrics, University of Pavia, Policlinico San Matteo IRCCS Foundation, Pavia, Italy; National Center for Immunobiologicals Control and Evaluation, Istituto Superiore di Sanità, Rome, Italy; Control of Communicable Disease Department, Institute of Public Health, Tirana, Albania; National Neurological Institute IRCCS Foundation C. Mondino, Pavia, Italy

**Keywords:** Poliovirus, Oral polio vaccine, Vaccine-associated paralytic poliomyelitis, X-linked agammaglobulinemia, Acute flaccid paralysis, Vaccine-derived poliovirus

## Abstract

**Background:**

Vaccine-associated paralytic poliomyelitis (VAPP) and immunodeficient long-term polio excretors constitute a significant public health burden and are a major concern for the WHO global polio eradication endgame.

**Case presentation:**

Poliovirus type 3 characterized as Sabin-like was isolated from a 5-month-old Albanian child with X-linked agammaglobulinemia and VAPP after oral polio vaccine administration. Diagnostic workup and treatment were performed in Italy. Poliovirus replicated in the gut for 7 months. The 5’ non coding region (NCR), VP1, VP3 capsid proteins and the 3D polymerase genomic regions of sequential isolates were sequenced. Increasing accumulation of nucleotide mutations in the VP1 region was detected over time, reaching 1.0 % of genome variation with respect to the Sabin reference strain, which is the threshold that defines a vaccine-derived poliovirus (VDPV). We identified mutations in the 5’NCR and VP3 regions that are associated with reversion to neurovirulence. Despite this, all isolates were characterized as Sabin-like. Several amino acid mutations were identified in the VP1 region, probably involved in growth adaptation and viral persistence in the human gut. Intertypic recombination with Sabin type 2 polio in the 3D polymerase region, possibly associated with increased virus transmissibility, was found in all isolates. Gamma-globulin replacement therapy led to viral clearance and neurological improvement, preventing the occurrence of persistent immunodeficiency-related VDPV.

**Conclusions:**

This is the first case of VAPP in an immunodeficient child detected in Albania through the Acute Flaccid Paralysis surveillance system and the first investigated case of vaccine associated poliomyelitis in Italy since the introduction of an all-Salk schedule in 2002. We discuss over the biological and clinical implications in the context of the Global Polio Eradication Program and emphasize on the importance of the Acute Flaccid Paralysis surveillance.

## Background

In 1988, the World Health Assembly launched the Global Polio Eradication Initiative. Live attenuated oral poliovirus vaccine (OPV) was selected by WHO for routine use and national immunization days. OPV might require several doses to induce immunity, but then it provides long-term protection against paralytic disease through durable humoral and mucosal immunity [1]. The use of OPV, however, is associated with some rare adverse events, including the occurrence of vaccine-associated paralytic poliomyelitis (VAPP) [2] and the emergence of vaccine-derived polioviruses (VDPVs) [3]. VAPP occurs at very low rates (∼1 case per 600,000 first-dose recipients) [4], and it is estimated that approximately 498 (range 255–1018) VAPP cases occur worldwide each year [2]. VAPP risk is 3000–7000 fold higher in persons with primary immunodeficiencies, notably agamma- and hypogammaglobulinemia, exposed to OPV [5]. In a small proportion of immunodeficient patients, Sabin strains can replicate in the intestine for several months or years [6–8], in contrast to the short (typically 3–4 weeks) period of replication in immunocompetent persons following their first OPV dose [9].

Because the poliovirus genome typically evolves at an average rate of 1 % per year, prolonged replication or circulation of viruses derived from OPV is recognized by the accumulation of genomic nucleotide substitutions [6]. VDPVs are operationally defined as vaccine-related isolates having >1 % nucleotide divergence from the corresponding type 3 or type 1 Sabin strain in the genome coding for the major capsid surface protein, VP1 (or >0.6 % in case of type 2 polioviruses). By contrast, OPV-like isolates have limited divergence from their parental OPV strains and are ubiquitous wherever OPV is used. The occurrence of immunodeficiency-related-VDPV (iVDPV) is typically consistent with at least 1 year of poliovirus replication since the administration of the first OPV dose. Nevertheless, evolution rates as fast as 2 %/year have been observed in the early phases of patients with iVDPV [10]. Since the introduction of OPV in 1961, more than 70 subjects with primary immunodeficiencies have been found excreting iVDPVs worldwide; the majority of these immunodeficiencies were detected after the onset of VAPP [1]. Type 2 iVDPVs are the most prevalent (64 %), followed by type 1 (21 %) and type 3 (15 %) [1]. By contrast, VAPP in immunocompetent OPV recipients and household contacts is most frequently associated with type 3 (71 %) followed by type 2 (26 %) poliovirus [5, 6].

Albania is considered polio free since 1997. The last polio outbreak of 1996 was caused by an imported wild type 1 poliovirus. Vaccination with OPV was maintained until March 2014, when a sequential schedule using inactivated polio vaccine (IPV) followed by OPV was adopted. The Acute Flaccid Paralysis (AFP) surveillance, established in 1997, has always met the WHO standard performance: two VAPP cases were detected until now, both affecting immunocompetent children after the first OPV dose [4].

Italy is considered polio-free since 1982, when the last indigenous wild polio cases occurred. Three imported wild polio cases from Iran, India and Libya (the last in 1988) were reported afterwards [11]. Since then, only rare VAPP cases (two of which were household contacts) have occurred in Italy [12, 13] before the adoption of Salk vaccine (IPV) in 2002 [14, 15]. The last VAPP case in Italy occurred in the year 2000 in a one-year old immunodeficient child who received three doses of OPV [16] and was detected through the AFP surveillance in force in Italy since 1997 according to the WHO guidelines [17].

We report the case of an Albanian infant affected by congenital agammaglobulinemia, who developed VAPP at the age of 5 months after receiving two doses of OPV in his country, and was then referred to Italy for further treatment. We isolated from sequential stool samples Sabin-like type 3 polioviruses, which accumulated 1 % of mutations in the VP1 viral genome in 7 months. We herein describe the genetic evolution of the poliovirus during the entire period of excretion, and discuss the implications of chronic virus excretion for the global polio eradication strategy.

This is the first case of VAPP in immunodeficient child detected in Albania through the AFP Surveillance system and the first iVDPV case investigated in Italy since the occurrence of the indigenous iVDPV case reported by Buttinelli and colleagues in 2003 [16].

## Case presentation

### Case report

XY, male, was born in Albania after a normal delivery from non-consanguineous parents in December 2013. The patient was vaccinated following the local schedule with bacille Calmette-Guérin (BCG) and hepatitis B vaccine at birth, and with pentavalent vaccine (diphtheria, pertussis, tetanus, hepatitis B and *Haemophilus influenzae* type b), 10-valent pneumococcal conjugate vaccine and trivalent OPV at the age of 2 and 4 moths. On June 2014, at the age of 5 months, he was hospitalized in Tirana University Hospital for the occurrence of acute flaccid quadriplegia with left facial paralysis and respiratory distress, 10 days after a self-limiting febrile enteritis. Blood investigations and C-reactive protein were normal. Cerebrospinal fluid (CSF) analysis demonstrated hypercellularity (196 cells/μL, 95 % lymphocytes), normal glucose concentration (53 mg/dl) and high proteinorrachia (100 mg/dl). CSF Gram stain and bacterial culture, and serology for *Herpes Simplex* type 1 and 2 were negative for recent infection. Cerebral magnetic-resonance imaging was normal. Electroneurography was consistent with axonal motor neuropathy. In the suspect of an inflammatory meningo-radiculitis, polyclonal intravenous immunoglobulins (IVIG) at the dose of 1 g/kg/day for two days were started, along with acyclovir and cefotaxime, followed by prompt respiratory function improvement. According to the AFP surveillance program, stools samples were collected and sent to the WHO National Polio Reference Laboratory in Tirana (Albania). This led to the isolation and identification of type 3 poliovirus, later characterized as Sabin-like by the WHO Regional Polio Reference Laboratory in Rome, Italy. Serum immunoglobulin levels after IVIG administration, were: IgG 1060 mg/dl (normal values 470–1230), IgA <6.7 mg/dl (normal values 21–145), IgM 5.7 mg/dl (normal values 47–175).

**Fig. 1:**
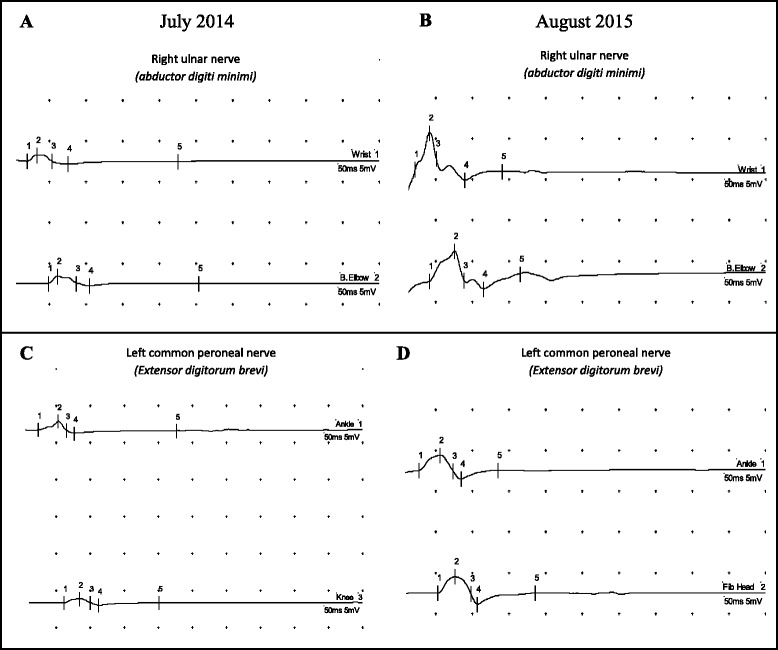
Electroneurography changes at baseline and after 1-year of follow-up. Electroneurography of the right ulnar (**a-b**) and left peroneal (**c-d**) nerves at the time of hospitalization in Italy (July 2014) (**a-c**) and at the 1-year follow-up (August 2015) (**b-d**). Notice the improvement of the composite motor action potentials (CMAP) amplitude in both the examined upper and lower limbs, which is consistent with axon regeneration

On July 2014, the patient was admitted to the Pediatric Clinic of San Matteo Hospital in Pavia (Italy) for further evaluation. Neurological assessment evidenced flaccid paralysis of the left lower limb and paresis of the right upper and lower limbs. Electroneurography (ENG) assessing motor and sensory nerve conduction and needle electromyography (EMG) was performed in proximal and distal muscles of upper and lower affected limbs, using Medelec Synergy EMG equipment (band-pass filter: 10–10,000 Hz) at the National Neurological Institute C. Mondino (Pavia, Italy). Findings were consistent with axonal motor neuropathy and muscular active denervation, with severe neurogenic signs, mainly at the right upper and left lower limbs (Fig. 1a and c). Immunological assessment proved absence of circulating B cells and inadequate immunoglobulin production after in vitro lymphocyte stimulation, with normal count and function of T lymphocyte subsets. These findings were consistent with vaccine-associated poliomyelitis in a patient with X-linked agammaglobulinemia. The congenital defect was confirmed by reduced in vitro expression of the BTK protein in blood leukocyte populations, and the identification of a causative de novo mutation [1922G > A] of the BTK gene. IVIG substitution therapy was started at 400 mg/kg/dose for 7 days and every three weeks thereafter, with strict serum immunoglobulins monitoring.

**Fig. 2:**
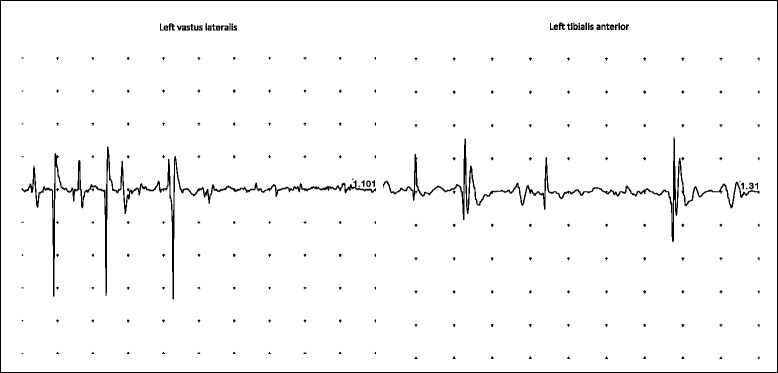
Electromyography traces of improved voluntary activity in the left lower limb at 1-year follow-up (August 2015). Signs of collateral motor reinnervation and axon regeneration in both the left vastus lateralis and tibialis anterior

All hygienic precautions for the possible spread of the virus were taken, according to the Centers for Disease Control and Prevention (CDC) guidelines for isolation precautions (2007) [18]. All the hospitalized patients in the Pediatric Unit at this time were vaccinated with either IPV or with OPV according to their vaccination schedules. We alerted all medical and non-medical staff, as well as the patient’s parents, to the potential risk for virus transmission. A physiotherapy program was started, and the child was discharged with signs of mild neurological improvement. The residual paralysis of the upper and lower limbs significantly improved with adequate physiotherapy at 1-year follow up. ENG/EMG performed on August 2015, 13 months after baseline, showed recovery of motor nerve conduction amplitudes and signs of muscular reinnervations (Figs. 1 and 2). Electroneurographic changes consistent with axonal regeneration are evidenced in Table 3.

The child’s parents expressed informed written consent for publication of all clinical data.

### Virus isolation and typing

Stools from the patient were processed according to the Polio Laboratory Manual, WHO [19] inoculating 0.5 ml of the 10 ml chloroform-extracted samples onto two RD and two L20B (selective for polioviruses) cell monolayers in 50 ml cell culture flasks. Samples negative at first isolation underwent two serial blind passages in both cell lines, and samples positive on RD cells were passaged on L20B for specific amplification of poliovirus. Stool specimens collected in June in Albania were processed at the WHO National Polio Reference Laboratory in Tirana (NRL); stools samples collected in Italy in July, September and thereafter were analysed at the WHO Regional Polio Reference Laboratory in Rome (RRL). Polio typing at the NRL was performed by monospecific anti-PV pooled sera RIVM/WHO kit (National Institute of Public Health and Environment, RIVM, Bilthoven, Netherlands in accordance with the WHO protocol) [19]. Typing at the RRL was obtained by real time reverse transcription PCR (rRT-PCR) with specific primers (CDC) [19].

### Intratypic differentiation and genome sequencing

Intratypic differentiation (ITD) between Sabin-like or non-Sabin-like polioviruses was performed by rRT-PCR with specific primers (CDC) [19]. Sequencing of the 5’NCR (nt 179 to 575), VP1 (nt 2479 to 3378), VP3 (nt 1948–2146) and 3D (nt 6134 to 6353) genomic regions, following rRT-PCR amplification, was performed for all type 3 polioviruses isolates: two from Albania (A1 and A2 June) and 4 from Italy (B1 and B2 July, C1 and C2 September) (Table 1). The ITD and sequencing of all strains were performed at the WHO Regional Polio Reference Laboratory in Rome, Italy.

**Table 1:** Collected samples and cell culture isolation

Specimen	Origin	Collection date		RD^a^	L20B^b^
A	ALBANIA	A1 11-06-14	A2 13-06-14	Positive (PV3)	Positive (PV3)
B	ITALY	B1 18-07-14	B2 21-07-14	Positive (PV3)	Positive (PV3)
C	ITALY	C1 11-09-14	C2 12-09-14	Positive (PV3)	Positive (PV3)
D	ITALY	D1 24-09-14	D2 24-09-14	Negative (3^rd^ passage)	Negative (3^rd^ passage)
E	ITALY	E1 24-10-14	E2 24-10-14	Negative (3^rd^ passage)	Negative (3^rd^ passage)
F	ITALY	F1 24-11-14	F2 24-11-14	Negative (3^rd^ passage)	Negative (3^rd^ passage)
G	ITALY	G1 16-12-14	G2 17-12-14	Negative (3^rd^ passage)	Negative (3^rd^ passage)
H	ITALY	H1 14-01-15	H2 14-01-15	Negative (3^rd^ passage)	Negative (3^rd^ passage)

Specimens A to H refer to the sequential collection of different stool samples from the immunodeficient patient. Type 3 poliovirus (PV3) was isolated from the first stool specimens (A1, A2) collected in Albania in June 2014, and from four stool samples collected in Italy until September 2014 (B1, B2, C1 and C2)

^a^ RD, Rhabdomyosarcoma cell line

^b^ L20B, Recombinant murine cell line specific for polioviruses

The amplified sequences of the 5’NCR, VP1, VP3 and 3D region were analyzed using sequencing software. The percentage of identity was determined on the number of nucleotide substitutions per site with respect to the reference Sabin type 3 strain. Amino acid sequence alignments were obtained using the software Sequencer [20]. Nucleotide sequence accession numbers for the reference Sabin type 3 and type 2 were AY184221.1 and AY184220. The nucleotide sequences of the 5’NCR, VP1, VP3 and 3D regions of polioviruses have been deposited in the GenBank database (accession numbers: KU708511-KU708514). We used the program Phyre2 to localize in the three-dimensional structure of the type 3 poliovirus the amino acid substitutions found in the capsid proteins [21].

### Neutralization assay

Serum samples were tested for their neutralizing activity against the three poliovirus serotypes at the RRL, Rome. Two fold dilutions of sera were incubated with equal volumes of Eagle’s MEM containing 100 TCID_50_ (tissue culture infectious dose) of Sabin poliovirus (types 1, 2 and 3). Plates were scored for cytopathic effect (CPE) on the third, fifth and seventh day. Neutralization titers were defined as the reciprocal of the highest dilution of serum yielding a 50 % reduction of CPE [22]. Seropositivity was defined as reciprocal titers of poliovirus neutralizing antibodies ≥8.

## Results

### Virus isolation

Type 3 polioviruses (PV3) were isolated from the first stool specimens (A1 and A2 June) collected in Albania and from stools samples (B1 and B2 July) and (C1 and C2 September) collected in Italy at S. Matteo Hospital. Stools collected thereafter were negative for poliovirus replication after three blind passages on L20B and RD cells cultures (Table 1). No other poliovirus or non-polio enteroviruses were isolated.

### Characterization of polio type 3 isolates and genomic analysis

All PV3 strains were characterized as Sabin-like by ITD. In order to study the genome evolution during replication, the RNA of six sequential strains (A1, A2, B1, B2, C1 and C2) was analyzed by nucleotide sequencing. The nucleotide differences of the strains with respect to the PV3 Sabin reference evidenced for all isolates a reversion (U > C) at position 472 of the 5’NCR in the domain V of the internal ribosome entry site (IRES). This mutation is known to occur very rapidly upon replication of the virus in the human gut, and plays an important role in the attenuated phenotype of poliovirus type 3 [23–25]. An additional mutation was found in all strains in the 5’NCR at nucleotide (nt) 292 (G > A), in the domain IV of the IRES. These mutations have been described in other PV3 and VDPVs isolates from immunodeficient patients [24, 26–28]. The mutations identified in the region that codes for the major capsid protein VP1 (nt 2477–3376) with respect to Sabin type 3 are reported in Table 2. In the first isolate (A1 June), mutations were located at position 150 (C > T), 161 (C > T) generating an amino acid substitution at amino acid (aa) 54 (Ala > Val), and at position 336 (T > C), 417 (C > T) and 846 (G > A). In the second isolate (A2 June), two additional mutations were found at position 192 (A > G) and 193 (G > A), this last one generating a replacement (Val > Ile) at aa 65. The isolate (B1 July) collected in Italy presented two further mutations at position 314 (T > C), which leads to an amino acid replacement at aa 105 (Met > Thr), and at position 774 (T > C). All nucleotide mutations and amino acid substitutions found in (B1 July) persisted during the entire period of excretion without further changes.

**Table 2:** Nucleotide and amino acid variation in the VP1 region

11/06/2014 ALB (A1)				13/06/2014 ALB (A2)				18/07/2014 ITA (B1)				21/07/2014 ITA (B2)				11/09/2014 ITA (C1)				12/09/2014 ITA (C2)			
NT		AA		NT		AA		NT		AA		NT		AA		NT		AA		NT		AA	
150	C > T			150	C > T			150	C > T			150	C > T			150	C > T			150	C > T		
161	C > T	54	A > V	161	C > T	54	A > V	161	C > T	54	A > V	161	C > T	54	A > V	161	C > T	54	A > V	161	C > T	54	A > V
				192	A > G			192	A > G			192	A > G			192	A > G			192	A > G		
				193	G > A	65	V > I	193	G > A	65	V > I	193	G > A	65	V > I	193	G > A	65	V > I	193	G > A	65	V > I
								314	T > C	105	M > T	314	T > C	105	M > T	314	T > C	105	M > T	314	T > C	105	M > T
336	T > C			336	T > C			336	T > C			336	T > C			336	T > C			336	T > C		
417	C > T			417	C > T			417	C > T			417	C > T			417	C > T			417	C > T		
								774	T > C			774	T > C			774	T > C			774	T > C		
846	G > A			846	G > A			846	G > A			846	G > A			846	G > A			846	G > A		

Nucleotide substitutions and the relative amino acid changes in the VP1 capsid region, in sequential isolates during the excretion period

*NT* nucleotide, *AA* amino acid

**Fig. 3:**
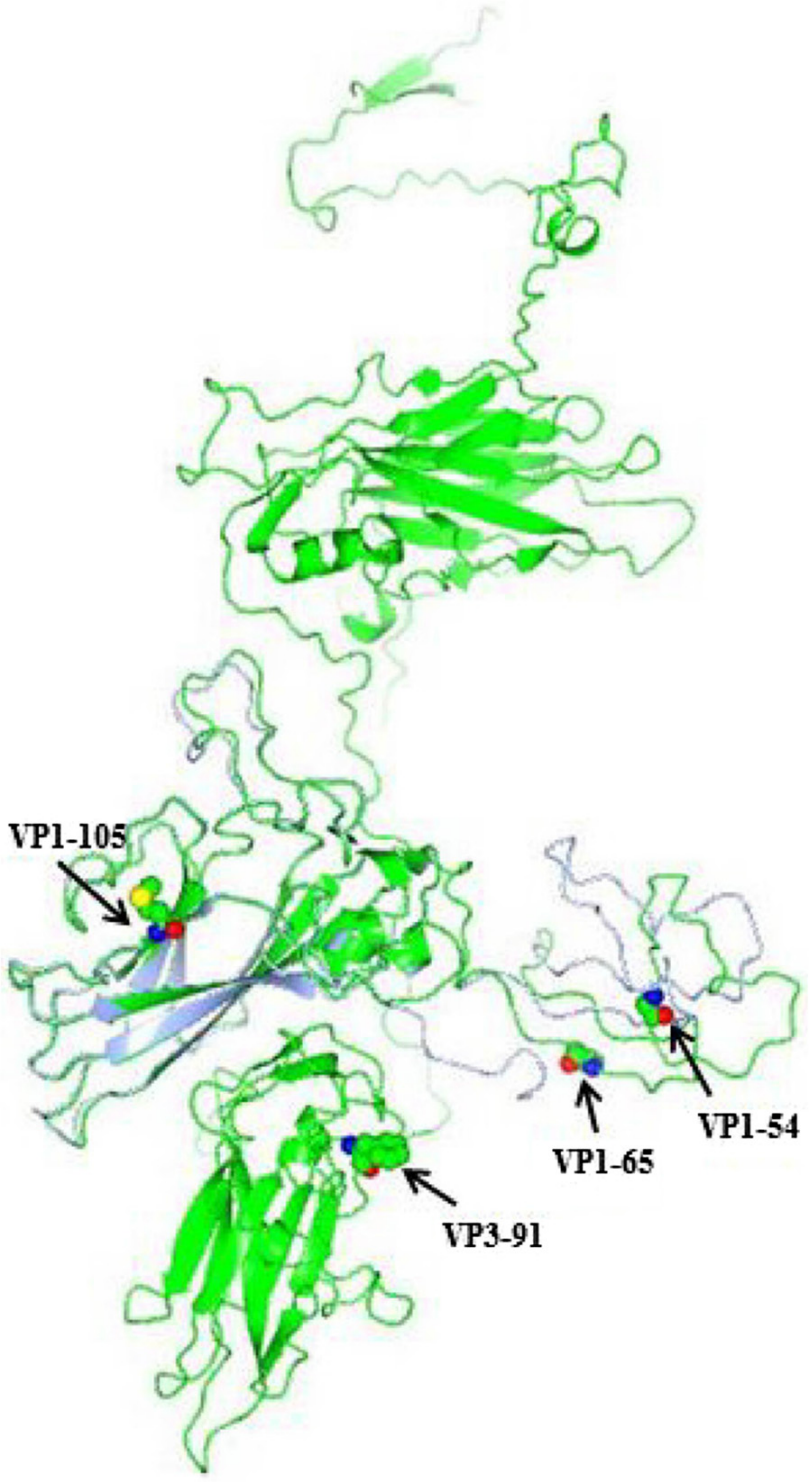
Ribbon diagram of the α-carbon trace of the Sabin type 3. Complete frontal view of the VP1-VP4 capsid proteins. Amino acid substitutions in the capsid proteins VP1 and VP3 found in the isolated poliovirus type 3 are highlighted

The evolution rate, expressed as a percentage of changes with respect to Sabin type 3, was rather constant during the excretion period, increasing from 0.5 % in the first isolate to 1 % in the last isolate (Table 2). This corresponds approximately to 1–2 changes per week. The sequencing of the VP3 region revealed in all PV3 isolates a mutation at nt 273 (T > C) causing an amino acid substitution at position 91 (Phe > Ser). This mutation is involved in the capsid assembly and is known to be correlated with the neurovirulent phenotype in type 3 polioviruses [29, 30]. The sequential amino acid substitutions in VP1 and VP3 mapped onto the three-dimensional X-ray crystallographic atomic coordinates of Sabin type 3 are shown in Fig. 3.

The sequencing of the 3D polymerase-coding region revealed an intertypic recombination Sabin 3/Sabin 2 in all isolated strains. The Sabin 2 genome encompassed the entire 3D region and no mutation was found with respect to the reference Sabin 2.

### Neutralizing antibodies

The two sera collected in September and December 2014 at San Matteo Hospital in Pavia had passive neutralizing antibody titers against all three PV: sample (C September) had a titer of 1:32 for PV1, 1:91 for PV2 and 1:91 for PV3, sample (G December) had a titer of 1:23 for PV1, 1:32 for PV2 and 1:64 for PV3. The WHO considers as protective a titer >1:8 [31].

## Conclusions

Sabin OPV vaccine has been adopted by the WHO for the Global Polio Eradication Initiative (GPEI) and decades of experience have shown that it is safe and effective in preventing poliomyelitis. However, it is known that attenuated viruses may revert to neurovirulent phenotypes upon replication in the human gut, rarely causing VAPP in immunocompetent individuals [2]. Immunodeficient persons vaccinated with OPV have a 3000–7000 time higher risk of developing VAPP, especially when affected by agammaglobulinemia or hypogammaglobulinemia [32]. Moreover, in immunodeficient subjects attenuated OPV viruses replicate in the gut and can persist for long periods [15, 33–35]. Excreted viruses rapidly accumulate mutations and genetic rearrangements, increasing the chance of reversion to neurovirulence and transmissibility. For these reasons, long-term excretors of poliovirus and immunodeficient patients are an additional concern for the WHO, as they could constitute a polio reservoir in the post-eradication era, and infect both unvaccinated and immunocompromised persons [8, 36, 37].

In this work, we report a detailed molecular analysis of six sequential type 3 polioviruses isolated from an Albanian child affected by congenital agammaglobulinemia who developed flaccid paralysis 1 month after receiving the second dose of OPV in his country. The ITD of the strains confirmed the Sabin-like characteristic of all PV3 isolates.

In middle- and high-income countries, VAPP occurs more frequently after receiving the first dose of OPV and can affect unvaccinated contacts. Poliovirus type 2 (Sabin or iVDPV) is the prevalent isolated serotype in immunodeficient individuals, while type 3 only counts for 15–32 % of isolations [1, 2, 38]. Our patient developed VAPP after receiving the second dose of OPV, at the age of 5 months. Interestingly, he had been breastfed during the first 4.5 months of life. Enteroviruses are cleared from the host mainly by antibody-mediated mechanisms. Secretory IgA can neutralize viruses by recognizing specific capsid antigens, reducing infectivity and promoting viral clearance [39, 40]. The patient’s mother had been vaccinated with OPV, which stimulates durable mucosal immunity through IgA secretion. We speculate that passive transfer of maternal IgA via breast-feeding could have played a role in limiting virus proliferation in the gut of our patient after the first dose of OPV. It is also known that the presence of anti-polio antibodies in the blood prevents the central nervous system (CNS) invasion [41]. In our case, we can assume that the physiological postnatal decreasing of maternal antibodies, associated with breast-feeding interruption in the absence of self secretory-IgA, led to a rapid viral replication in the gut, and subsequently invasion of the CNS, causing VAPP at the age of 5 months.

After the first virus isolation in Albania in June, stool samples were monthly collected during his hospitalization in Italy and analyzed by the WHO RRL for polio in Rome to verify poliovirus persistence and genetic evolution. In all samples collected until September 2014 (ALB A1, A2, and ITA B1, B2, C1, C2) a poliovirus type 3 was isolated. Samples collected thereafter were negative for polio and other enteroviruses (Table 1). This reveals, in contrast with other reported cases [24, 34], a quick clearance of poliovirus in our patient, with a complete eradication after 3 months of IVIG administration. Therapy was well tolerated, without side effects, and led to objective clinical and neurophysiological improvement (Table 3). No antivirals were administered in our case. The rapid clearance of the virus in this subject is likely due to the timely administration of IVIG at high doses with strict IgG concentration monitoring (compared to the less useful monthly administration of fresh frozen plasma therapy [24] or IVIG at lower doses [27]). A further explanation could be the presence of high antibody titers against type 3 in the specific sera used for the replacement therapy that would allow a better neutralization. However, there’s still little knowledge on IVIG use in VAPP, possibly because most of the cases have been reported in the past, before the availability of polyclonal injectable immunoglobulins, and recently only in low-income countries where IVIG are too expensive or still not available.

**Table 3:** Electroneurographic values at baseline and at the 1-year follow-up

Nerve	July 2014			August 2015		
	Latency (ms)	Amplitude (mV)	Velocity (m/s)	Latency (ms)	Amplitude (mV)	Velocity (m/s)
Right ULNAR						
1. Wrist	2,00	0,9		2,20	4,5	
2. Elbow	4,90	0,9	39,7	4,20	3,8	62,5
Right MEDIAN						
1. Wrist	3,05	1,4		2,90	1,0	
2. Elbow	5,30	0,7	40,0	5,70	1,2	41,1
Left COMMON PERONEAL						
1. Ankle	2,40	1,2		2,75	1,9	
2. Fibular Head	5,05	0,8	41,5	5,30	2,1	47,1

Comparison of the electroneurographic values at the time of hospitalization in Italy (July 2014) and after 1 year (August 2015) with evidence of significant improvement in the motor potentials amplitude of both the examined upper and lower limbs

The lack of isolation of poliovirus type 1 or 2 Sabin-like strains might be related to reduced replication of these serotypes in the human gut. We can nonetheless hypothesize a greater passive protection against these two serotypes conferred by maternal antibodies.

Sequencing of the 5’NCR and VP3 regions evidenced a U > C mutation at nt 472 in the domain V of the IRES and at the amino acid residue 91 (Phe > Ser): both correlate in PV3 with reversion of the attenuated phenotype, confirming their importance for neurovirulence and possibly for adaptation and replication in the gut [24, 25, 40]. The additional mutation in 5’NCR nt 292 (G > A) in the domain IV of the IRES was also found in other PV3 isolated from iVDPV and VAPP cases [24, 25] but its role in conferring neurovirulence has not been demonstrated. Mutations in VP1 quickly increased over time reaching 1 % genome variation in the samples (ITA C1 and C2) collected in September 2014. The strains were still Sabin-like, but the percentage of accumulated nucleotide mutations was very close to the value >1 % that defines an iVDPV.

Viruses replicating in immunodeficient patients tend to select characteristic mutations at different times during the infection, suggesting that specific selection pressures may operate in the gut over time. The amino acid substitutions at position 105 (Met > Thr) and 54 (Ala > Val) of VP1 have already been described by Martin and colleagues in a PV3 strain isolated from an immunodeficient patient [24]. Amino acid VP1 105 is located at the north rim of the canyon in the hydrophobic pocket, and plays a role for the uncoating of the virus. The substitution 54 (Ala > Val) in VP1 acts as a suppressor of the temperature sensitivity and attenuation phenotype (the capacity of growth at elevated temperatures is indeed typical of wild poliovirus strains) [42]. Other mutations that are known to be involved in growth adaptation, viral persistence and increased neurovirulence, such as sequence variation at nucleotide 2493 in VP1[43, 44], were not found in our patient.

Finally, sequencing of the 3D polymerase revealed the presence of an intertypic recombination with type 2 Sabin (recombinant PV3/PV2 Sabin) in all isolates. This is an interesting finding, even though similar recombinations have already been described in healthy vaccinees, iVDPV and VAPP cases [33, 45].

The global prevalence of immunodeficient subjects with chronic polio infection is unknown and if asymptomatic they remain undetected. This poses a risk in the context of the GPEI endgame. Therefore, the detection of chronic iVDPV excretors in all countries and the development of antivirals to eradicate chronic infections is a WHO priority. Sequence properties of circulating VDPV (cVDPV) strains are distinguishable from iVDPV strains excreted by chronic infected persons. The comparison between the mutational patterns found in iVDPV and cVDPV can predict the origin of the anonimous VDPV (aVDPV) strains isolated during the environmental surveillance. This remains crucial for epidemiological interpretation because it may alert for the presence of a chronic iVDPV excretor in the community [3]. Environmental surveillance will be implemented by the GPEI in low income countries since it proved to be very sensitive in detecting cVDPV and iVDPV strains [46]. In this sense the mutations found in our study can add knowledge to the definition of iVDPVs genetic pattern.

OPV plays a key role in the eradication of wild poliovirus and is still used in regions where wild poliovirus is not definitively eradicated or with high risk of polio outbreak. Occurrence of cVDPV outbreaks and iVDPV cases and the potential spread of highly neurovirulent poliovirus strains to the environment has recently led the WHO to state that OPV usage will globally be discontinued soon after the certification of global eradication [3, 6, 24]. The endgame plans for the GPEI actually include the synchronic replacement of the trivalent Sabin live-attenuated oral poliovaccine (tOPV) with the bivalent bOPV (PV1,3) as a first step to prevent the more frequent cVDPV type 2 outbreaks. The subsequent withdrawal of all OPV use and the maintenance of high immunization coverages with IPV will protect against imported wild polioviruses and prevent cVDPV outbreaks and new iVDPV infections [47]. As Albania did in 2014, most low income countries are incorporating at least 1 dose of IPV into routine childhood immunization schedules. The production of less expensive inactivated Salk IPV vaccine based on the Sabin strains vaccines is encouraged by the WHO for environmental safety and a large-scale use [47]. Because many high-income countries have replaced OPV with IPV, the VAPP burden is currently concentrated in lower-income countries. The planned universal introduction of IPV will also substantially decrease the global VAPP cases [2]. However, it is important that all countries maintain high quality of AFP surveillance and improve the current laboratory typing methods to better differentiate between VDPVs and wild poliovirus strains.

This is the first case of poliomyelitis and long-term excretion from an immunodeficient patient detected in Albania through the Acute Flaccid Paralysis surveillance system. Due to the high levels of immigration across the Mediterranean sea, Italy, Albania and other Mediterranean countries remain at risk of importing wild poliovirus from endemic areas as well as Sabin and neurovirulent VDPVs from countries currently using OPV. Moreover, since IPV vaccine (adopted in Italy since 2002) may not elicit a consistent mucosal immunity, silent transmission of neurovirulent poliovirus might occur through IPV-immunized individuals, favouring possible infection of unvaccinated subjects or children receiving delayed vaccination [22, 48, 49]. For these reasons an Active AFP surveillance is in force at national level as well as an environmental surveillance in seven large Italian cities with high immigration rate [45]. Immunodeficient patients have been monitored for several years in Italy and no poliovirus long-term excretors have been detected [50]. In Albania this screening has not been performed, but in the future environmental surveillance and monitoring of immunodeficient persons will be implemented.

No antivirals are currently available to interrupt poliovirus excretion in immunodeficient subjects. The capsid-binding drug pleconaril that prevents poliovirus cell-entry has been tried in a reported case by our group with successful interruption of viral excretion when combined with IVIG treatment [16]. Developing effective drugs/treatments is a WHO priority and research is ongoing in this field.

## Abbreviations

AA (or aa), amino acid; AFP, acute flaccid paralysis; CNS, central nervous system; CRP, C-reactive protein; cVDPV, circulating VDPV; ENG/EMG, electroneurography and electromyography; GPEI, global polio eradication initiative; IPV, inactivated polio vaccine (Salk); ITD, intratypic differentiation; iVDPV, immunodeficiency-associated VDPV; IVIG, polyclonal intravenous immunoglobulins; L20B, recombinant murine cell line; NRL, National Reference Laboratory for poliovirus; NT (or nt), nucleotide; OPV, oral polio vaccine (Sabin); PV, poliovirus; PV1, poliovirus type 1; PV2, poliovirus type 2; PV3, poliovirus type 3; RD, rhabdomyosarcoma cell line; RRL, Regional Reference Laboratory for poliovirus; rRT-PCR, real-time reverse transcription polymerase chain reaction; VAPP, vaccine-associated paralytic poliomyelitis; VDPV, vaccine-derived poliovirus
